# Neural evidence for phonologically based language production deficits in older adults: An fMRI investigation of age‐related differences in picture‐word interference

**DOI:** 10.1002/brb3.660

**Published:** 2017-03-15

**Authors:** Avery A. Rizio, Karlee J. Moyer, Michele T. Diaz

**Affiliations:** ^1^ Department of PsychologyThe Pennsylvania State UniversityUniversity ParkPAUSA

**Keywords:** cognitive aging, language, magnetic resonance imaging, phonology impairment, semantics

## Abstract

**Introduction:**

Older adults often show declines in phonological aspects of language production, particularly for low‐frequency words, but maintain strong semantic systems. However, there are different theories about the mechanism that may underlie such age‐related differences in language (e.g., age‐related declines in transmission of activation or inhibition).

**Methods:**

This study used fMRI to investigate whether age‐related differences in language production are associated with transmission deficits or inhibition deficits. We used the picture‐word interference paradigm to examine age‐related differences in picture naming as a function of both target frequency and the relationship between the target picture and distractor word.

**Results:**

We found that the presence of a categorically related distractor led to greater semantic elaboration by older adults compared to younger adults, as evidenced by older adults’ increased recruitment of regions including the left middle frontal gyrus and bilateral precuneus. When presented with a phonologically related distractor, patterns of neural activation are consistent with previously observed age deficits in phonological processing, including age‐related reductions in the recruitment of regions such as the left middle temporal gyrus and right supramarginal gyrus. Lastly, older, but not younger, adults show increased brain activation of the pre‐ and postcentral gyri as a function of decreasing target frequency when target pictures are paired with a phonological distractor, suggesting that cuing the phonology of the target disproportionately aids production of low‐frequency items.

**Conclusions:**

Overall, this pattern of results is generally consistent with the transmission deficit hypothesis, illustrating that links within the phonological system, but not the semantic system, are weakened with age.

## 1 Introduction

Age‐related declines in language production are a common and frustrating aspect of aging. These declines include slower word retrieval (Mortensen, Meyer, & Humphreys, 2006), increased pauses in speech (Vousden & Maylor, 2006), and increased word retrieval failures, such as the tip‐of‐the‐tongue phenomenon (Burke, Mackay, Worthley, & Wade, 1991). Because a multitude of behavioral studies indicate that semantic processes and general knowledge are generally well maintained with advanced age (e.g., Burke & Peters, 1986; Stine & Wingfield, 1994; Verhaeghen, 2003; Waters & Caplan, 2005), the causes of word retrieval failures lie in other aspects of language (e.g., phonological processes) or cognition (e.g., inhibitory deficits, general slowing, working memory declines). The goal of the current study was to examine the neurological basis of these age‐related differences in language production using a picture‐word interference (PWI) paradigm.

The PWI task provides the opportunity to explore the ways in which successful language production can be hindered or facilitated. When a target picture is presented in conjunction with a semantically related word, naming latencies are slowed as the distracting information activates a network of conceptually related items, thereby increasing the time required to select the target name (Glaser & Dungelhoff, 1984; Lupker, 1979; Rayner & Posnansky, 1978). In contrast, presenting a phonologically related word speeds target naming, facilitating the activation of phonemes that are shared by both the distracter and target. The timing of the distractor's presentation in relation to the target has provided insight into the time course of the specific stages of language production. Lexical selection occurs early in the production processes, as it can be impeded by presenting a semantic distractor prior to or at the onset of the target picture. As a result, the degree of semantic interference and target response latencies are increased (e.g., Glaser & Glaser, 1989). In contrast, phonological retrieval occurs at a later stage in the production time course, as evidenced by the finding that phonological facilitation can be increased by presenting the distractor at or after the onset of the target picture (e.g., Schriefers, Meyer, & Levelt, 1990).

The neurological basis of both semantic interference and phonological facilitation has been explored in younger adults, converging on the importance of both temporal and frontal regions. Semantic distractors elicit greater activation in the middle temporal gyrus when compared to nonword distractors (de Zubicaray, Wilson, McMahon, & Muthiah, 2001) and phonological distractors (Diaz, Hogstrom, et al., 2014). This region is recognized as being part of the ventral stream of speech processing (Hickok & Poeppel, 2007), and has been shown to specifically support semantic processing (Tyler, Moss, & Jennings, 1995; Wright, Stamatakis, & Tyler, 2012). Additional research (Indefrey & Levelt, 2004) has suggested that activation in this region is associated with lexical selection, suggesting that the presence of a semantic distractor during production increases selection demands. Although the superior frontal gyrus and anterior cingulate are not typically included in models of language production, previous research has shown their engagement during semantic interference (de Zubicaray et al., 2001). Activation in the right superior frontal gyrus has been interpreted to reflect increased inhibitory control and attempts to suppress irrelevant information (e.g., Rizio & Dennis, 2013; Wylie, Foxe, & Taylor, 2008), while the anterior cingulate cortex is believed to monitor for the presence of competition (e.g., Barch, Braver, Sabb, & Noll, 2000; Botvinick, Nystrom, Fissell, Carter, & Cohen, 1999; Kerns et al., 2004). As such, it is possible that competition between a semantic distractor and the target requires the recruitment of inhibitory control regions to mitigate the effects of interference. Taken together, the neural correlates of semantic interference are largely observed as increases in activation in regions that support a combination of semantic processing, lexical selection, inhibitory control, and competition monitoring.

With respect to phonological facilitation, most studies have converged on the importance of the superior temporal gyrus (STG) and supramarginal gyrus (SMG), but the direction of the effects associated with this activation is less clear. With respect to language production in general, the STG and superior temporal sulcus support phonological processing during both speech perception and speech production (e.g., Buchsbaum, Hickok, & Humphries, 2001; Hickok & Poeppel, 2007), while the supramarginal gyrus supports phonological working memory (Paulesu, Frith, & Frackowiak, 1993). The earliest studies of picture‐word interference reported decreased STG activation for phonological distractors relative to unrelated distractors (de Zubicaray & McMahon, 2009; de Zubicaray, McMahon, Eastburn, & Wilson, 2002), with this reduction in activation suggesting that phonological distractors produce the equivalent of priming effects. In support of this interpretation, Graves, Grabowski, Mehta, and Gupta (2008) reported that repetition suppression effects in the STG were specifically related to more efficient phonological access. However, other studies have reported an opposite pattern of activation, reporting that the presence of a phonological distractor results in *increased* activation. Increased activation has been observed in the left STG and SMG when compared to both semantic distractors (Diaz, Hogstrom, et al., 2014) and unrelated distractors (Abel et al., 2009), while activation of the bilateral middle temporal gyrus (MTG) has also been reported for phonological compared to unrelated distractors (Abel, Dressel, Weiller, & Huber, 2012). Overall, it appears as though target repetition may strongly influence whether phonological processing is associated with increased or decreased activation. Studies that use a target picture more than once have reported decreases in activation during the presentation of phonological distractors (de Zubicaray & McMahon, 2009), while studies that only present a target once typically report increased activation for phonological trials, relative to other conditions (e.g., Abel et al., 2009; Diaz, Hogstrom, et al., 2014). Thus, while the presence of a phonological distractor during a PWI task appears to facilitate access to the phonology of the target, additional research is needed to determine whether this effect is linked to increases or decreases in activation of regions that support this processes (i.e., SMG, STG, MTG).

While advances have been made in understanding the brain systems that support language production during the presentation of distracting information in younger adults, similar investigations in older adults have been limited to behavioral research. Despite the overall age invariance of comprehension (Burke & Peters, 1986; Stine & Wingfield, 1994; Verhaeghen, 2003; Waters & Caplan, 2005), older adults often experience deficits in language production. For example, older adults tend to be slower when naming objects (for review see Mortensen et al., 2006), generate more off‐topic speech (Arbuckle, Nohara‐LeClair, & Pushkar, 2000), have more pauses in their speech (Vousden & Maylor, 2006), and experience more tip‐of‐the‐tongue states than younger adults (Burke et al., 1991). Multiple theories have been developed to account for the variety of cognitive changes experienced by older adults, but two in particular provide the most comprehensive explanations for changes in language processes. The inhibition deficit theory (IDT) suggests that older adults are less able to control what information enters their conscious awareness, making it more likely that they will be distracted by additional information (Hasher, Stoltzfus, Zacks, & Rypma, 1991). This theory predicts that older adults should process distracting information to a greater extent than younger adults, resulting in greater semantic interference *and* phonological facilitation. The transmission deficit hypothesis (TDH) posits that the strength of connections within the semantic and phonological language systems weakens with age (MacKay & Burke, 1990). Because the semantic system is highly interconnected, the weakening of a single connection between the lexical node for a word and a specific semantic representation is unlikely to result in retrieval failure. In this way, older adults may not show age‐related declines on semantic tasks because the multiple connections between semantic representations and a lexical node can help to compensate for the effects of transmission deficits. In contrast, because the phonological system has fewer connections (i.e., only between the sounds within a word), and because there is a one‐to‐one mapping between a phonological representation and a lexical node, transmission deficits are much more likely to result in retrieval failure (Burke & Shafto, 2004). Additionally, the TDH posits that low‐frequency items are most vulnerable to transmission deficits, because lack of frequent or recent use weakens connections that would aid retrieval. While low‐frequency words are typically associated with slower naming latency even in younger adults (e.g., Jescheniak & Levelt, 1994), older adults’ production is theorized to be even more strongly affected by frequency because they already experience age‐related weakened connections throughout the phonological system (Taylor & Burke, 2002). Only one study has investigated age‐related differences in production during interference, which reported that older adults exhibit greater semantic interference effects than younger adults, but show equivalent behavioral effects for phonological distractors (Taylor & Burke, 2002). These results are more consistent with the TDH than they are with the IDT. Older adults may experience greater semantic interference than younger adults in part because they have larger semantic networks. However, the phonological distractor may serve as a prime for the target picture, thereby reducing potential transmission deficits by momentarily strengthening the connection between lexical and phonological nodes (Taylor & Burke, 2002). While this priming aided retrieval for older adults, making their performance comparable to younger adults, older adults did not perform *better* than younger adults, as would be predicted by the inhibition deficit theory (Taylor & Burke, 2002). The results of this single study (i.e., momentary reduction in transmission deficits by phonological distractors), combined with the TDH's premise that production deficits in older adults can be exacerbated by low lexical frequency, suggests that the facilitatory effects of phonological distractors should be highest for low‐frequency items. This hypothesis, however, has not yet been empirically tested.

Although the neural correlates of age‐related differences in phonological facilitation and semantic interference have not yet been explored, studies have investigated the way in which regions that support language production differ across the lifespan. For example, many studies have reported that frontal activation, particularly within the left inferior frontal gyrus, is largely age‐invariant (e.g., Destrieux et al., 2012; Meinzer et al., 2009; Shafto, Stamatakis, Tam, & Tyler, 2010). Production tasks also frequently elicit increased activation in additional regions for older compared to younger adults, although these increases are not always associated with maintained performance. For example, during a verbal fluency task, older adults showed more activation in the right inferior frontal gyrus than younger adults, but this activity was negatively correlated with the number of items produced (Meinzer et al., 2009, 2012; but see Wierenga et al., 2008). Similarly, during both semantic and phonological judgment tasks that required covert naming of pictures, older adults showed increased activation relative to younger adults in regions including the left middle and superior frontal gyri, and left middle and superior temporal gyri (Diaz, Johnson, Burke, & Madden, 2014). This activation was not, however, correlated with behavioral performance, suggesting that these increases in activation did not aid production (Diaz, Johnson, et al., 2014). Taking into account the possibility that task difficulty may strongly influence patterns of brain activation in older adults, Persson, Lustig, Nelson, and Reuter‐Lorenz (2007) demonstrated that at low levels of task difficulty, both older and younger adults deactivated brain regions that were not necessary for successful language production. When faced with a more difficult task, however, older adults were less able to suppress these task‐irrelevant activations (Persson et al., 2007). Overall, although older adults activate regions within the frontal and temporal lobes that are typically associated with language production, they do so to a different extent when compared to younger adults. Moreover, these increases in activation outside of the traditional left‐hemisphere language network are not always beneficial to performance.

The primary goal of the current study was to examine the neural correlates of age‐related differences in language production in the presence of distracting information. While this question has not yet been addressed in the literature, the aforementioned studies provide insight into possible patterns of brain activation. The IDT predicts that older adults should process all distractors to a greater extent than younger adults. In this way, older adults should exhibit greater phonological facilitation and semantic interference, at both the behavioral and neural levels, than younger adults. At the behavioral level, inhibition deficits should result in age‐related increases in trials in which the distractor word is produced instead of the target name, and slower reaction times for semantic and unrelated trials. Additionally, if inhibition deficits contribute to age differences in language production, older adults should show deficits in the ability to recruit inhibitory control regions such as the superior frontal gyrus and anterior cingulate when compared to younger adults, and as a consequence, should also exhibit neural evidence of greater semantic interference. Because semantic interference has been linked to increased activation in regions including the middle temporal gyrus, IDT predicts that older adults should exhibit age‐related increases in this region. Moreover, older adults should also process phonologically related distractors to a greater extent than younger adults, resulting in age‐related differences in regions associated with phonological priming, such as the superior temporal gyrus, and again, reduced recruitment of inhibitory control regions. If target stimuli are not repeated throughout the experiment, phonological processing should be observed as increases in activation in these regions. Unlike the IDT's focus on regions that support cognitive control, the TDH predicts differences in activation associated with core language‐related regions. Specifically, older adults should experience greater semantic interference effects than younger adults due to larger semantic networks, but phonological facilitation effects that are equal to that of younger adults. In this way, older adults should exhibit more activation than younger adults in regions associated with semantic processes, such as the middle temporal gyrus and inferior frontal gyrus, but there should be no age‐related differences in regions associated with phonological distractors.

Additionally, we wished to explore the effect of target frequency on brain activation. If the presence of a phonological distractor aids production in older adults by strengthening the connections between lexical and phonological nodes, as posited by the TDH, then facilitation should be strongest for low‐frequency targets. While younger adults can experience transmission deficits, they do not experience the same weakening of connections within the phonological system that older adults do. For this reason, we predict that the difference in facilitation between high‐ and low‐frequency targets should be significantly greater for older compared to younger adults.

## 2 Methods

### 2.1 Participants

A total of 20 younger adults between the ages of 18 and 31 (females = 10, mean age = 23.7) and 20 older adults between the ages of 60 and 79 (females = 15, mean age = 67) participated in the experiment. All were healthy, right‐handed, native English speakers who were not fluent in a second language. All had normal or corrected‐to‐normal vision, as measured by the Freiburg Visual Acuity and Contrast Test (Bach, 1996), and no one reported a history of neurological or psychological disorders. All participants scored at least 27 on the Mini Mental State Examination (MMSE; Folstein, Folstein, & McHugh, 1975). Before the MRI session, each participant completed assessments to determine handedness and language history, and performed a battery of psychometric and neuropsychological tests to measure aspects of cognition including speed, inhibition, working memory, and language (see Table 1). All participants provided written informed consent, and all experimental procedures were approved by the Institutional Review Board at the Pennsylvania State University.

**Table 1:** Participant demographic and neuropsychological testing information

	Younger adults	Older adults
	Mean (SD)	Mean SD)
Demographic information		
N	20	20
Age^a^	23.7 (4.32)	67.25 (6.16)
Gender (M/F)	10/10	5/15
Education (years)	16.6 (2.72)	16.75 (2.17)
Neuropsychological testing		
MMSE	29.25 (0.91)	28.65 (1.04)
Vocabulary	53.25 (8.13)	56.25 (5.5)
Immediate recall	12 (1.86)	11.25 (1.68)
Delayed recall	11.2 (2.26)	9 (2.49)
Verbal fluency	66.2 (15.5)	67.84 (15.18)
Simple speed	264.8 (36.17)	284.65 (42.74)
Complex speed^a^	282.96 (28.4)	345.17 (77.94)
Digit span forward	7.15 (1.09)	7.15 (1.31)
Digit span backward	5.3 (1.30)	4.8 (1.2)
Digit symbol^a^	1291.29 (260.66)	1832.3 (328.77)
Stroop effect^a^	12.35 (30.67)	92.87 (65.91)
Nonverbal working memory^a^	0.76 (0.07)	0.66 (0.12)
Author recognition^a^	14.60 (8.76)	35.20 (14.76)
Magazine recognition^a^	12.70 (6.63)	23.65 (7.34)

a Scores for which a significant age difference exists, *p *<* *.05.

### 2.2 Stimulus materials and procedure

**Figure 1:**
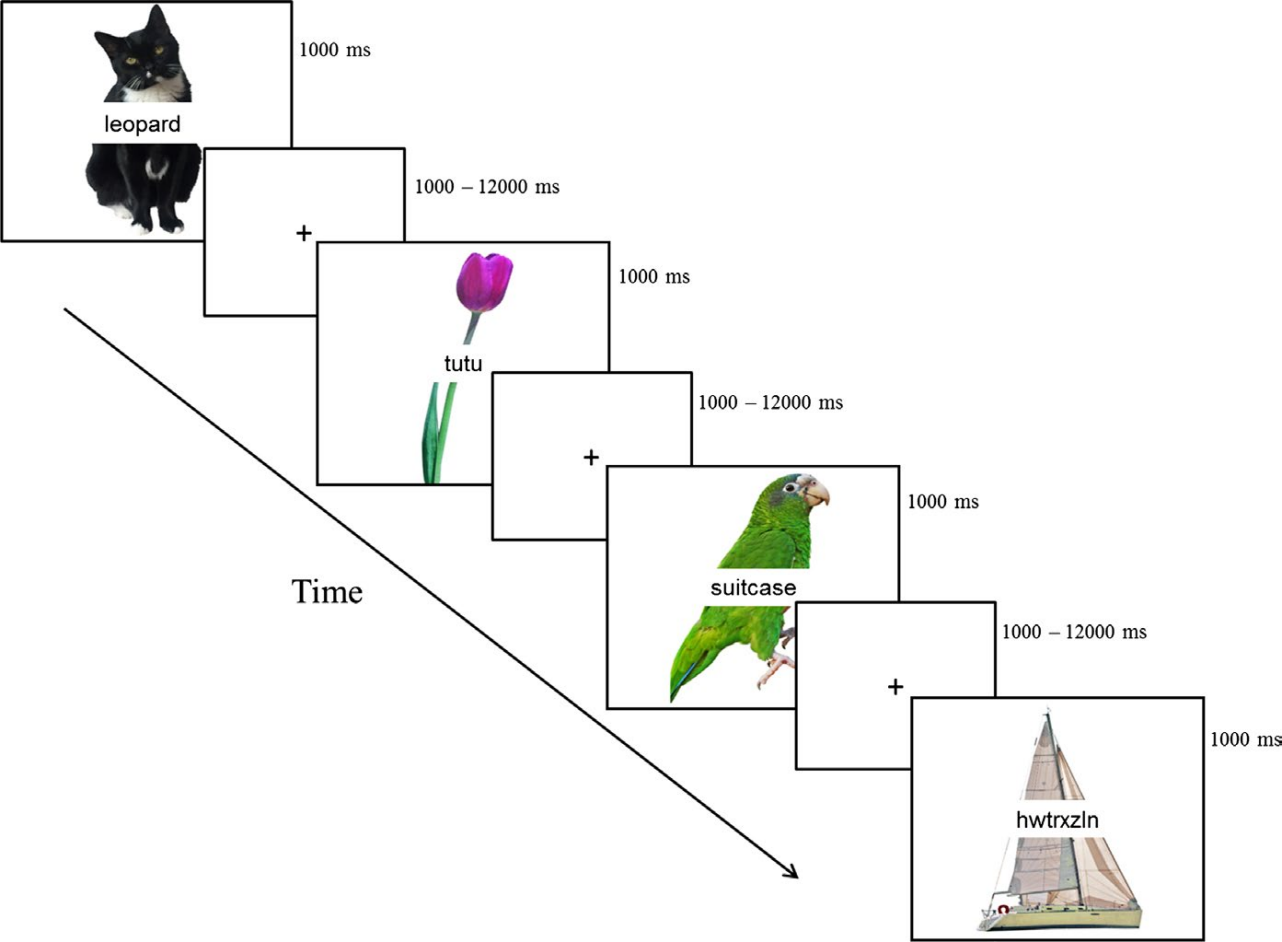
Task design. Examples of each of the four distractor conditions: categorical, phonological, unrelated, and nonword. Correct responses to the target pictures are “cat,” “tulip,” “parrot,” and “sailboat,” respectively

Stimuli consisted of 240 colored images, 60 unique items per condition, which were presented with a written distractor word superimposed (see Figure 1). Pictures were taken from two normed picture databases (Brodeur, Guerard, & Bouras, 2014; Moreno‐Martinez & Montoro, 2012). Based on naming data provided by the aforementioned databases, the average name agreement of the selected images was 72%. Images depicted common concrete objects from a variety of categories such as animals, clothing, food, and household items. Frequencies for all stimuli were obtained from the English Lexicon Project (Balota et al., 2007), using the log‐transformed HAL frequency, which is based on the Hyperspace Analogue to Language corpus (see Table 2 for all word characteristics). The average log frequency of the target picture name was 7.48 (*SD* = 1.91), and ranged from 2.30 to 12.44 (log frequencies of all words in the database range from 0 to 17). The lexical frequency range of the items was intentionally broad to assess the influence of picture frequency on naming.

**Table 2:** Stimuli characteristics as a function of condition

	Log frequency	Word length	Target–Distractor categorical relatedness	Target–Distractor orthographic similarity
	Mean (SD)	Mean (SD)	Mean (SD)	Mean (SD)
Word type				
Target picture	7.48 (1.91)	6.47 (2.07)	—	—
Categorical distractor	7.43 (1.66)	6.78 (1.92)	6.16 (1.37)	0.10 (0.09)
Phonological distractor	7.70 (1.86)	6.13 (1.80)	1.36 (0.40)	0.49 (0.11)
Unrelated distractor	7.51 (1.92)	6.28 (1.81)	1.31 (0.36)	0.10 (0.89)
Nonword distractor	—	6.18 (1.76)	—	0.08 (0.08)

Distractor words were concrete nouns, and belonged to one of four categories: categorical, phonological, unrelated, and nonword. No distracter word was used more than once. There was no statistical difference between the frequency of the distractor words across conditions and target words [*F*(2, 717)  = 1.31, *ns*], nor were there differences in the distractor word length [*F*(3, 956)  = 0.42, *ns*] (see Table 2). Moreover, distractor word frequency was not significantly correlated with target word frequency. Four distractors (one for each condition) were created for each of the 240 target images. Four different stimuli lists were created, each with a different combination of target and distractor pairs. In this way, participants were presented with a target image only once in order to avoid picture repetition, but *across* participants, each target was paired with each of the four distractor types to control for any differential item effects (e.g., picture complexity, lexical characteristics, etc.). Categorically related distractors were semantically, but not associatively, related to the target picture. Categorical relatedness was measured on a scale of one (not at all categorically related) to seven (very categorically related) by 47 younger adults who did not participate in the MRI session. There was a significant difference in judgment of categorical relatedness across the four conditions [*F*(2, 717)  = 2545.81, *p *<* *.001]. Specifically, distractors that had been selected by the experimenters as categorically related to the target were judged by participants to be more related to the target than distractors in the phonological condition [*t*(478)  = 21.80, *p *>* *.001] and the unrelated condition [*t*(478)  = 42.69, *p *>* *.001]. Both phonological and unrelated distractors were judged to be equally semantically unrelated to the targets [*t*(478)  = 1.26, *ns*].

Phonologically related distractors shared at least the two initial phonemes with the target image. Phonological relatedness was first determined through the use of the Carnegie Mellon Pronouncing Dictionary (www.speach.cs.cmu.edu/cgi-bin/cmudict) to ensure that words were precisely matched on both initial sound and stress. Phonological relatedness was also quantified by calculating the orthographic similarity (OS) of target and distractor pairs. OS was calculated in NIM (Guasch, Boada, Ferre, & Sanchez‐Casas, 2013) using the following equation: Orthographic Similarity = Graphemic Similarity between the target and distractor/Graphemic Similarity of the target with itself. OS values can range from 0 (no similarity) to 1 (identical). Because phonological distractors were matched only on the first two phonemes, the average OS value for phonological‐target pairs was 0.49 (*SD* = 0.11). There was a significant difference among the OS values for target–distractor pairs [*F*(2, 717)  = 1328.25, *p *<* *.001]: target–phonological pairs had significantly greater OS values than target–categorical pairs [*t*(478)  = 41.79, *p *>* *.001], target–unrelated pairs [*t*(478)  = 43.32, *p *>* *.001], and target–nonword pairs [*t*(478)  = 54.00, *p *>* *.001].

Unrelated distractors were neither categorically nor phonologically related to the target. Nonword distractors consisted of random consonant strings that did not start with the same letters as the target.

Each trial consisted of a target picture and a distractor word that was superimposed over the center of the target picture (Figure 1). Each picture was presented on a white background (7 × 5.5 inches). Pictures were constrained to either 7’’ wide or 5.5’’ tall to regularize the size of the objects without distorting the aspect ratio. Distractors were presented in Courier New 18 point font. Target pictures and distractor words were presented simultaneously (i.e., SOA = 0) for 1 s. Participants were instructed to name the target picture, but ignore the distractor word, and to respond as quickly as possible while still responding accurately. A fixation cross was presented between each stimulus presentation [interstimulus interval (ISI) range = 1–12 s, average ISI = 4 s]. ISIs were optimized with Optseq2 (Dale, 1999). Participants were instructed that they had both the duration of the target presentation, as well as the duration of the ISI, to make their response. Trials were randomized so that no more than three of the same distractor condition appeared in a row. Each of four runs (315 s) began and ended with the presentation of a fixation cross.

Prior to scanning, participants practiced overt picture naming while minimizing head movement (in a simulation scanner). Practice trials included pictures and distractors that were not used during the experiment. Participants were not familiarized with the target pictures used during the scanning session prior to entering the MRI. In the scanner, overt verbal responses were recorded and filtered using an MR‐compatible fiber optic microphone system (Optoacoustics Ltd., Or‐Yehuda, Israel).

### 2.3 Acquisition of MRI data

Imaging data were acquired using a 3 T Siemens Prisma Fit MRI scanner with a 20‐channel head coil. T‐1 weighted anatomical images were collected using a magnetization‐prepared rapid acquisition gradient echo (MP RAGE) sequence with an anterior to posterior phase encoding direction, and a 7 ms echo spacing. Spatial parameters were set such that FOV = 256 mm^2^, matrix = 256 mm^2^, with a voxel size of 1 mm^3^. 160 contiguous slices, 1 mm thick, were acquired in ascending order. Timing parameters were set such that TR = 2300 ms, TE = 2.28 ms; TI = 900 ms. The flip angle = 8˚, and fat suppression was not used.

An advanced shim was applied before the first functional run. Functional images were collected using an echo‐planar imaging (EPI) sequence with an anterior to posterior phase encoding direction and a 0.49 ms echo spacing. Spatial parameters were set such that FOV = 240 mm^2^; matrix = 80 mm^2^, with a voxel size of 3 mm^3^. Forty‐one contiguous slices were acquired in interleaved order. Timing parameters were set such that TR = 2500 ms; TE = 25.0 ms. A total of 126 volumes per run were collected for the fMRI analysis. Two additional volumes were acquired and deleted at the beginning of each functional run to reach steady state equilibrium. These volumes were not included in the fMRI analyses. The flip angle = 90˚, and fat saturation was used.

### 2.4 Behavioral data analysis

Recordings from the scanner session were transcribed for naming accuracy. Trials were marked as correct if the participant provided the exact name for the target image (e.g., cat for cat), the plural form of the target image (e.g., socks for sock), or an abbreviated form of the target, as long as the first two phonemes of the response and target matched (e.g., rhino for rhinoceros). Accuracy rates for each condition (categorical, phonological, unrelated, and nonword) were calculated by dividing the total number of correct responses by the total number of targets in that condition (60). Trials were market as incorrect if any other answer was provided, or if no answer was provided. These errors were coded as one of four types: no response, alternative response (e.g., kitty for cat; robe for bathrobe), incorrect response (e.g., golf club for hoe), or instances in which the participant read the distractor word instead of naming the target.

The percentage of trials in which a participant made a specific type of error was calculated by dividing the number of trials in which that type of error occurred by the total number of trials (240), and multiplying the resulting quantity by 100.

Response latencies for each trial were calculated using customized Praat scripts, which marked word onsets by searching the audio file for places in which the pitch deviated from the pitch of the filtered background scanner noise. These onsets were then verified by manual inspection of both the auditory and visual speech stream. Target picture onsets, as recorded by E‐Prime, were subtracted from the response onsets to yield a measure of response latency for each trial, which was coded by distractor type. Response latency was measured in milliseconds (ms) and only accurate trials were included in our statistical analyses of response latency.

### 2.5 fMRI data analysis

A quality assurance protocol assessed acquired images for the number of potentially clipped voxels, mean signal fluctuation to noise ratio (SFNR), and per‐slice variation (Glover et al., 2012). Functional and anatomical images were visually inspected for artifact and signal drop‐out. Non‐brain tissue of the anatomical images was removed using Optimized Brain Extraction for Pathological Brains (optiBET: Lutkenhoff et al., 2014). All additional processing and analyses were conducted through FSL version 5.0.4, with FEAT (fMRI expert analysis tool) version 6.0 (Smith et al., 2004; Woolrich, Behrens, Beckman, Jenkinson, & Smith, 2004). Preprocessing steps included motion correction (FSL MCFLIRT), slice timing correction, spatial smoothing (FWHM = 5 mm), high‐pass filtering, coregistration, and normalization. Functional images were first coregistered to the participant's own brain‐extracted anatomical image and then registered to MNI space using an FSL template (MNI 152 T1 2 mm). A double‐gamma hemodynamic response function was used to model BOLD signal for each event.

Neuroimaging analyses focused on functional activation during target picture naming as a function of distractor condition. Only trials in which the participant accurately named the target (as described above) were included in analyses. Analyses were first conducted on participants’ individual runs, and were then conducted across runs. These analyses were combined across participants in a group analysis using the FMRIB local analysis of mixed effects (FLAME 1). A group analysis combining all participants, across age group, was performed, in addition to separate group analyses for younger and older adults. Within FSL, comparisons were made between distractor conditions, focusing on differences in activation between the phonological, categorical, and unrelated conditions (e.g., phonological > categorical; phonological > unrelated). All analyses employed a whole‐brain approach, with significant activations determined through a two‐step approach. First, statistically significant clusters were identified using a z threshold of 2.3. P values for these clusters were then calculated and corrected for multiple comparisons according to Gaussian Random Fields (GRF) theory, so that only those smaller than *p *<* *.05 corrected were retained. Age‐related differences in activation were also analyzed through additional *t*‐tests in order to investigate contrasts between distractor conditions (e.g., phonological > categorical) that also differed significantly between age groups. In addition to the thresholding and correction for multiple comparisons described above, a conjunction analysis was incorporated with the group difference analyses such that these results were masked with the results of the individual group contrast map. This procedure ensures that age differences were driven by increases in activation in the primary group of interest (e.g., younger adults in a younger > older group comparison), rather than by deactivations in the other group (e.g., older adults).

Additional analyses used the same procedure described above, but included a parametric design to explore activation within a given contrast that increased in accordance with either increasing or decreasing target word frequency. For these analyses, each individual trial was weighted by the mean‐centered frequency of the target picture. A second set of parametric analyses employed a weighting that was calculated by subtracting the distractor frequency from the target frequency, in order to explore regions of the brain that increased in activation as the difference between target and distractor frequency increased.

## 3 Results

### 3.1 Behavioral results

#### 3.1.1 Frequency effects

To verify the well‐established frequency effect, we conducted a series of correlations to determine whether the frequency of the target image had an effect on the time with which participants named them. To minimize the influence of the distractor, in this analysis, we examined target frequencies associated with the nonword distractors. When considering all participants collapsed across age groups, there was a significant negative correlation between target frequency and response latency, *r*(39)  = −.224, *p *<* *.005, such that the longest response latencies were associated with the lowest frequency target images. We next investigated this relationship for each individual age group. A negative correlation between target frequency and younger adult response latency was statistically significant, *r*(19)  = −.205, *p *<* *.005, such that the longest response latencies were associated with the lowest frequency target images. A negative correlation between target frequency and older adult response latency was not statistically significant, *r*(19)  = −.105, *ns*. However, a Fisher r‐to‐z transformation indicated that the correlation coefficients that represent the strength of the relationship between target frequency and response latency were not significantly different between younger and older adults (*p *=* *.28).

#### 3.1.2 Naming accuracy

**Figure 2:**
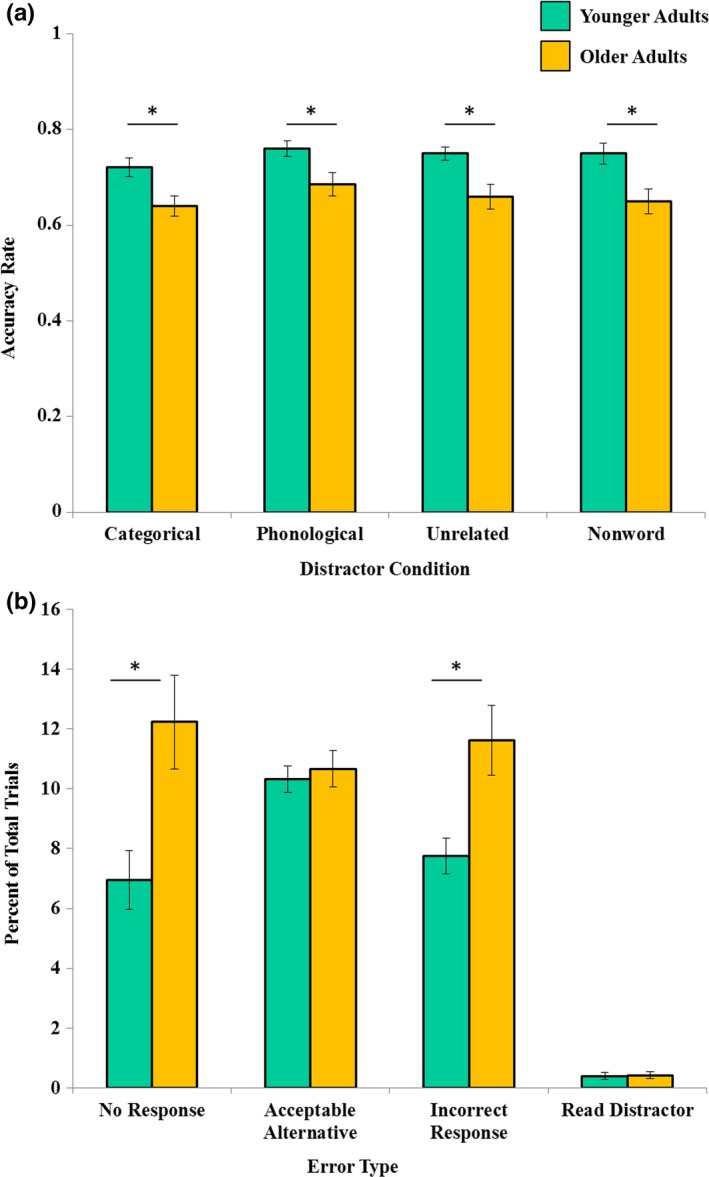
Naming accuracy and errors made during PWI task. (a)Younger adults were significantly more accurate in picture naming than older adults, across all distractor conditions. (b) Older adults made significantly more no responses and incorrect responses than younger adults during picture naming. Both groups had very few instances in which they read the distractor word instead of naming the target

A 4 (distractor condition: categorical; phonological; unrelated; nonword) × 2 (age group: younger; older) ANOVA was conducted to examine the effect of distractor conditions and age group on target naming accuracy. The main effect of distractor condition was not significant [*F*(3, 38)  = 2.25, *ns*] indicating that participants’ target naming accuracy was not affected by the type of distractor presented. There was a main effect of age group [*F*(1, 38)  = 14.29, *p *<* *.005], as younger adults’ rate of naming accuracy (*M *=* *0.75, *SE* = 0.013) was significantly higher than that of the older adults (*M *=* *0.66, *SE* = 0.019). There was no significant distractor condition by age interaction [*F*(2, 38)  = 0.23, *ns*] (see Figure 2a). Collapsed across age groups, the percent naming accuracy obtained in the current study (70.5%) is on par with the target pictures’ percent name agreement (72%) that was reported in the picture database norming studies (Brodeur et al., 2014; Moreno‐Martinez & Montoro, 2012). Moreover, as noted in the methods section, these accuracies reflect our strict accuracy criteria. Older and younger adults produced an additional 10.67% and 10.31%, respectively, of responses that we classified as ‘acceptable alternatives’. But due to the constraints of the PWI task, responses with alternative onset phonemes could not be included in the analyses.

Because older adults were significantly less accurate in target naming than younger adults, we conducted an additional analysis to explore potential age differences in the type of errors that participants produced. Errors were coded as one of four types: no response, acceptable alternative, incorrect, or distractor (see method section for additional description). The percentage of total trials for which each error type occurred was calculated, and then submitted to a 4 (error type: no response; alternative; incorrect; distractor) × 2 (age group: younger; older) ANOVA. There was a significant main effect of error type, *F*(3, 38)  = 59.95, *p *<* *.005. Follow‐up *t*‐tests revealed that errors caused by the reading of the distractor (*M *=* *0.41%, *SE* = 0.08) occurred significantly less frequently than errors caused by producing no response [*M *=* *9.59%, *SE* = 0.93; *t*(39)  = 8.99, *p *<* *.005], an acceptable alternative [*M *=* *10.49%, *SE* = 0.38; *t*(39)  = 27.18, *p *<* *.005], or an incorrect response [*M *=* *9.69%, *SE* = 0.66; *t*(39)  = 12.88, *p *<* *.005].

As revealed by the accuracy analysis, the main effect of age was significant [*F*(1,38)  = 19.31, *p *<* *.005], such that when averaging across error type, younger adults (*M *=* *6.35%; *SE* = 0.32) had a significantly lower percentage of errors than older adults (*M* = 8.73%, *SE* = 0.43).^1^ The error type by age interaction was also significant [*F* (3, 38)  = 4.45, *p *<* *.05]. Follow‐up *t*‐tests indicated that older adults (*M* = 12.23%, *SE* = 1.57) had significantly more no responses than younger adults [(*M* = 6.96%; SE = 0.99) *t*(39)  = 2.85, *p *<* *.05]. Likewise, older adults (*M* = 11.63%, *SE* = 1.16) had significantly more incorrect response than younger adults [(*M* = 7.75%; SE = 0.61) *t*(39)  = 2.96, *p *<* *.05]. There were no age differences, however, when comparing the percentage of trials in which an acceptable alternative was provided [*t*(39)  = 0.47, *ns* OA: *M* = 10.67%; *SE* = 0.61; YA: *M* = 10.31%; *SE* = 0.44], or when the distractor word was read instead of the target [*t*(39)  = 0.13, *ns* OA: *M* = 0.42%; *SE* = 0.12; YA: *M* = 0.40%; *SE* = 0.12] (see Figure 2b).

#### 3.1.3 Response latency

**Figure 3:**
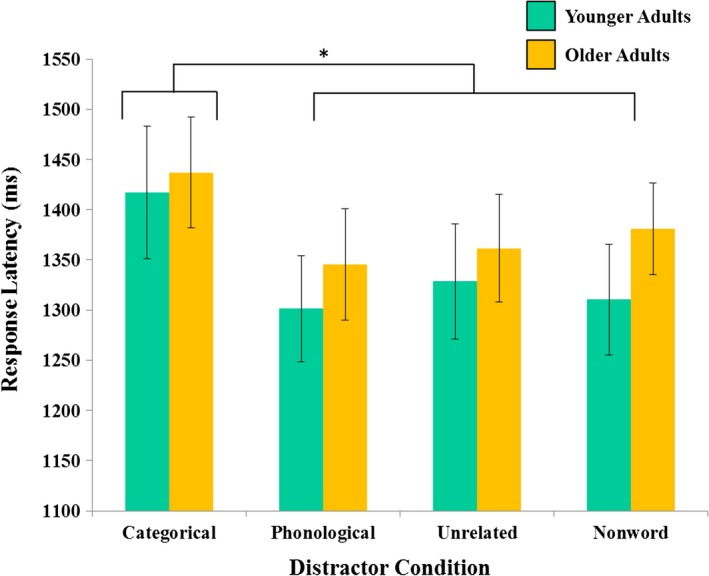
Naming latencies during PWI task. Targets paired with categorical distractors were named significantly more slowly than targets paired with any other type of distractor. No age differences in response latency were observed

To investigate the effects of distractor condition and age on response latency, a 4 (distractor condition: categorical; phonological; unrelated; nonword)  × 2 (age group: younger; older) ANOVA was conducted. There was a significant main effect of distractor condition, *F* (3, 38)  = 16.84, *p *<* *.001. Follow‐up *t*‐tests indicated that response latencies for target naming with semantic distractors (*M* = 1427.09 ms, *SE* = 44.28) was significantly slower than response latency with phonological [*M* = 1323.45 ms, *SE* = 39.29; *t*(39)  = 5.92, *p *<* *.005], unrelated [*M* = 1344.32 ms, *SE* = 40.09; *t*(39)  = 4.66, *p *<* *.005], and nonword [*M* = 1,346.32 ms, *SE* = 36.66; *t*(39)  = 4.54, *p *<* *.005] distractors. No other differences between distractor conditions were significant. There was no main effect of age group [*F* (1, 38) =0.289, *ns*], as response latencies for younger adults (*M* = 1339.34 ms, *SE* = 58.36) and older adults (*M* = 1381.26 ms, *SE* = 51.74) were not significantly different. Finally, the distractor condition by age interaction was not significant, *F* (3, 38)  = 0.96, *ns* (see Figure 3). Although RTs did not differ, older and younger adults may still differ in variability and overall speed. To address these concerns, we also conducted an analysis of RT using z‐transformed values following the recommendation of Faust, Balota, Spieler, & Ferraro, 1999. This analysis revealed the same pattern of results: a significant main effect of condition only, with no significant main effect of age group or condition × age group interaction.

Because age differences in response latency were not observed, a follow‐up study was conducted. Twenty younger adults were recruited to participate in exchange for course credit. These participants completed the same picture‐word interference task, with the same set of instructions and timing parameters. The only methodological difference was that these participants completed the task while seated in front of a computer in a testing booth, rather than in the MRI scanner. A 4 (distractor condition: categorical; phonological; unrelated; nonword)  × 2 (testing location: scanner; booth) ANOVA was conducted to investigate the possibility that younger adults who completed the task in the scanner were slower in their naming than would have been expected. A significant main effect of distractor condition [*F*(3, 38)  = 20.91, *p *<* *.005] revealed the same pattern of results demonstrated above, as response latencies for target naming with semantic distractors (*M* = 1224.00 ms, *SE* = 36.95) was significantly slower than response latencies with phonological [*M* = 1135.80 ms, *SE* = 29.82; *t*(39)  = 6.41, *p *<* *.005], unrelated [*M* = 1156.53 ms, *SE* = 32.75; *t*(39)  = 5.16, *p *<* *.005], and nonword [*M* = 1148.64 ms, *SE* = 31.73; *t*(39)  = 5.27, *p *<* *.005] distractors. The main effect of testing group was also significant [*F*(1, 38)  = 29.16, *p *<* *.005], as participants who completed the task in the scanner had significantly slower response latencies (*M* = 1339.34 ms, *SE* = 58.36) than participants who completed the task outside of the scanner (*M* = 993.15 ms, *SE* = 26.53). There was no significant distractor condition by testing group interaction [*F*(3, 38)  = 2.61, *ns*], indicating that while the younger adults who completed the task inside the scanner were slower overall, they exhibited the same effect of distractor type on naming latency as the participants who completed the task outside the scanner.

#### 3.1.4 Head movement

To assess the potential influence of the overt naming task on head motion, we conducted a 5 (functional run: naming run 1; naming run 2; naming run 3; naming run 4; resting state run)  × 2 (age group: younger; older) ANOVA. In this analysis, the dependent variable was millimeters (mm) of head movement. There was no main effect of functional run, [*F*(4, 38)  = 1.78, *ns*], indicating that runs in which participants performed overt naming (*M* across task runs = 0.21 mm, *SE* = 0.02) were not associated with significantly more head movement than the resting state run (*M* = 0.17 mm, *SE* = 0.02). There was a significant main effect of age [*F*(1, 38)  = 6.65, *p *<* *.05], such that head movement for younger adults (*M* = 0.17 mm, *SE* = 0.02) was significantly less than that of older adults (*M* = 0.23 mm, *SE* = 0.02). However, we note that the overall amount of motion, for both younger and older adults, was well below recommended standards of ½ ‐ 1 voxel (Poldrack, Mumford, & Nichols, 2011), given our voxel size of 3 mm^3^. Finally, the functional run by age interaction was not significant, [*F*(4, 38)  = 0.8, *ns*], suggesting similar patterns of head movement for both groups.

### 3.2 Neuroimaging results

#### 3.2.1 Picture naming in the presence of a categorical distractor

**Figure 4:**
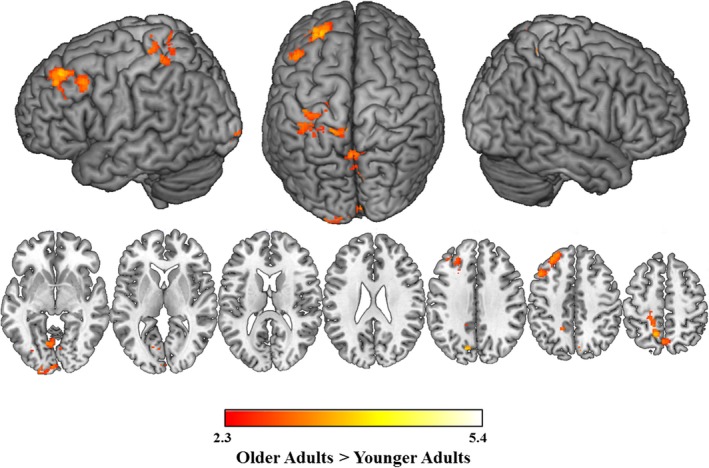
Age differences in semantic interference. Regions in which older adults elicited greater activation than younger adults during picture naming with a categorical distractor when compared to a phonological distractor. Slices are depicted in increments of 10, starting at z = −5 and ending at z = 55

When combining both younger and older adults, the presence of categorical distractors elicited greater activation than unrelated distractors in the bilateral middle frontal gyrus, bilateral middle temporal gyrus, and middle precuneus. Because no significant age differences were observed, we present only the combined results (see Table 3). We also compared the categorical distractor condition to the phonological distractor condition. When combining both younger and older adults, the presence of categorical distractors elicited greater activation than phonological distractors in the left lateral occipital cortex and cerebellum. Younger adults did not exhibit any activation for this comparison, and older adults elicited significantly greater extents of activation compared to younger adults in the left middle frontal gyrus, bilateral superior parietal lobe, bilateral precuneus, and bilateral lingual gyrus (see Table 3; Figure 4). Individual group patterns of activation are reported in the supplemental materials.

**Table 3:** Target naming with a categorical distractor

	H	Coordinates			Voxels	z value
		x	y	z		
Categorical > Unrelated						
All Participants						
Middle frontal gyrus	Right	40	8	48	1533	5.6
Superior frontal gyrus	Right	16	12	64		
Middle frontal gyrus	Left	−38	2	58	1271	4.72
Precentral gyrus	Left	−38	−6	58		
Middle temporal gyrus	Right	68	−34	−4	604	3.92
Middle temporal gyrus	Left	−68	−42	0	1211	4.51
Inferior temporal gyrus	Left	−52	−62	−24		
Precuneus	Middle	−6	−68	46	9282	4.3
Angular gyrus	Right	58	−52	42		
Lateral occipital cortex	Right	40	−58	48		
Precuneus	Middle	0	−68	50		
Younger > Older						
No activation differences						
Older > Younger						
No activation differences						
Categorical > Phonological						
All participants						
Cerebellum	Left	−52	−62	−26	1609	4.01
Posterior cingulate	Middle	−6	−38	8		
Lingual gyrus	Middle	4	−76	−10		
Occipital fusiform gyrus	Left	−14	−90	−22		
Lateral occipital cortex	Left	−32	−66	6	510	4.29
Younger > Older						
No activation differences						
Older > Younger						
Frontal pole, Middle frontal gyrus	Left	−28	38	46	382	3.64
Middle frontal gyrus	Left	−34	6	40	16	3.08
Posterior cingulate gyrus	Middle	−4	−40	28	22	2.87
Superior parietal lobe	Left	−42	−44	62	56	2.9
Superior parietal lobe	Left	−30	−50	68	19	2.58
Precuneus	Right	12	−50	60	35	3.23
Precuneus	Left	−12	−54	52	438	3.94
Precuneus	Middle	−2	−64	54	66	3.11
Precuneus	Left	−12	−74	36	75	3.69
Lingual gyrus	Right	18	−50	−8	13	2.86
Lingual gyrus	Right	22	−66	−6	279	3.25
Lingual gyrus	Right	10	−66	−10	29	2.92
Lingual gyrus	Right	8	−72	−2	19	2.82
Lingual gyrus	Left	−10	−84	−12	40	2.64
Occipital fusiform gyrus	Left	−32	−76	−6	17	2.57
Cuneus	Right	10	−80	38	27	3.3
Cerebellum	Left	−20	−48	−24	413	3.35

#### 3.2.2 Picture naming in the presence of a phonological distractor

**Figure 5:**
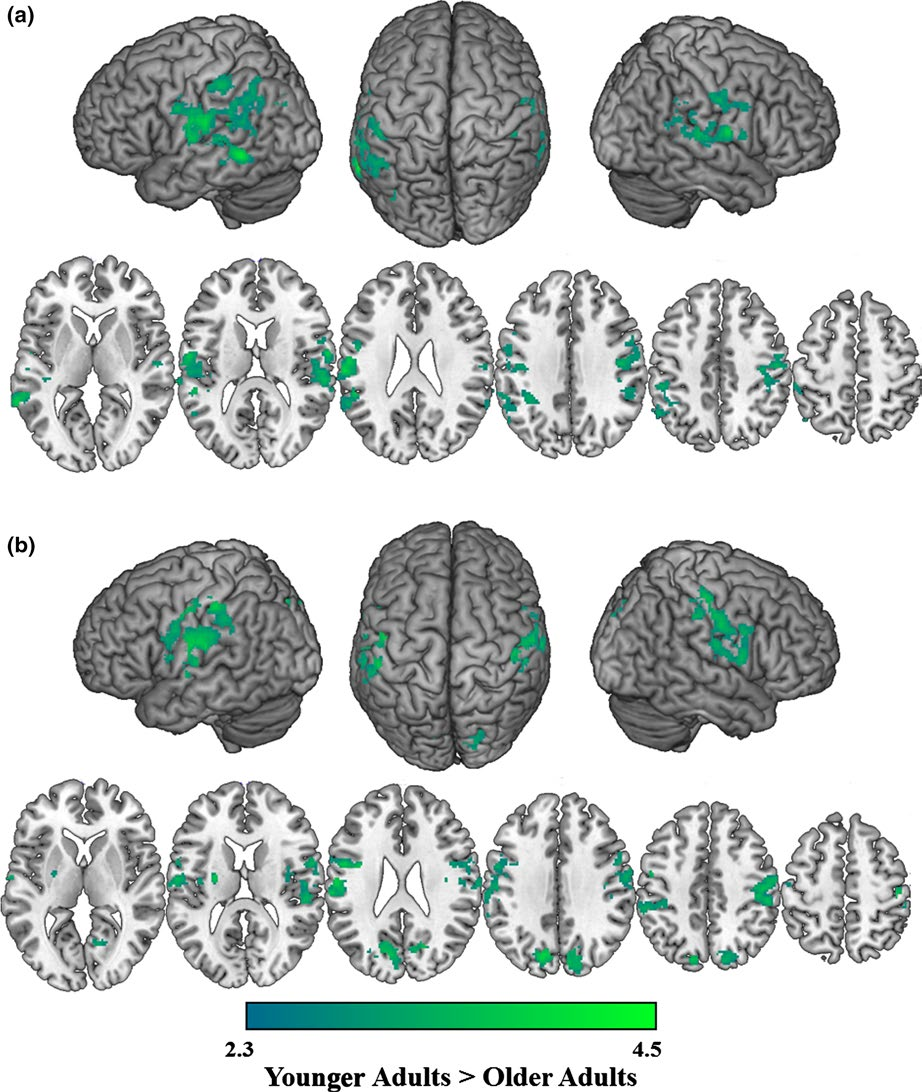
Age differences in phonological facilitation. Regions in which younger adults elicited greater activation than older adults for (a) phonological compared to unrelated distractors and (b) phonological compared to categorical distractors. Older adults did not elicit greater activation then younger adults for either contrast. Slices are depicted in increments of 10, starting at z = 5 and ending at z = 55

When combining both younger and older adults, the presence of phonological distractors elicited greater activation than unrelated distractors in the right angular gyrus and left superior parietal cortex. Age comparisons showed that compared to older adults, younger adults elicited greater activation in the right postcentral gyrus, right supramarginal gyrus, and bilateral middle temporal gyrus for phonological as compared to unrelated distractors (see Table 4; Figure 5a). We also examined brain activation of the phonological condition compared to the categorical condition. When combining both younger and older adults, the presence of phonological distractors elicited greater activation than categorical distractors in the right Heschl's gyrus. Age comparisons revealed that younger adults elicited greater activation than older adults for this contrast in regions including the bilateral central opercular cortex, which extended into the right insula, and left putamen, the bilateral postcentral gyrus, which extended into the bilateral precentral gyrus, bilateral precuneus, which extended into bilateral cuneus, and the right lingual gyrus (see Table 4; Figure 5b). Individual group patterns of activation are reported in the supplemental materials.

**Table 4:** Target naming with a phonological distractor

	H	Coordinates			Voxels	z value
		x	y	z		
Phonological > Unrelated						
All participants						
Angular gyrus	Right	38	−54	48	2919	4.51
Supramarginal gyrus	Right	66	−46	34		
Lateral occipital cortex	Right	52	−58	52		
Lateral occipital cortex	Right	34	−60	52		
Superior parietal lobe	Left	−36	−58	54	2539	4.21
Supramarginal gyrus	Left	−58	−36	52		
Angular gyrus	Left	−58	−56	44		
Younger > Older						
Postcentral gyrus	Right	62	−6	18	608	3.69
Postcentral gyrus	Right	44	−16	36	610	3.25
Supramarginal gyrus	Right	72	−34	28	24	2.91
Middle temporal gyrus	Right	54	−44	10	25	3.03
Middle temporal gyrus	Left	−64	−48	0	2486	4.09
Older > Younger						
No activation differences						
Phonological > Categorical						
All Participants						
Heschl's gyrus	Right	54	−8	4	557	3.49
Putamen	Right	32	−2	0		
Insula	Right	40	−6	8		
Central opercular cortex	Right	58	−16	18		
Younger Adults > Older Adults						
Central opercular cortex	Right	38	−10	22	80	3.17
Insula	Right	36	−8	16		
Central opercular cortex	Left	−40	−12	18	28	2.78
Putamen	Left	−26	−10	16	77	3.57
Postcentral gyrus	Left	−56	−12	22	1526	3.63
Precentral gyrus	Left	−48	−10	48		
Postcentral gyrus	Right	44	−22	54	1168	3.4
Precentral gyrus	Right	54	2	20		
Supramarginal gyrus	Right	52	−28	44		
Lingual gyrus	Right	14	−62	−2	121	3.35
Precuneus	Right	16	−76	40	532	3.41
Cuneus	Right	18	−68	24		
Precuneus	Left	−14	−76	36	463	3.73
Cuneus	Left	−10	−72	22		
Older Adults > Younger Adults						
No activation differences						

#### 3.2.3 Picture naming in the presence of an unrelated distractor

Compared to both phonological and categorical distractors, neither younger nor older adults elicited greater activation during picture naming in the presence of unrelated distractors.

#### 3.2.4 Effects of target frequency: increasing brain activation associated with decreasing target frequency

**Figure 6:**
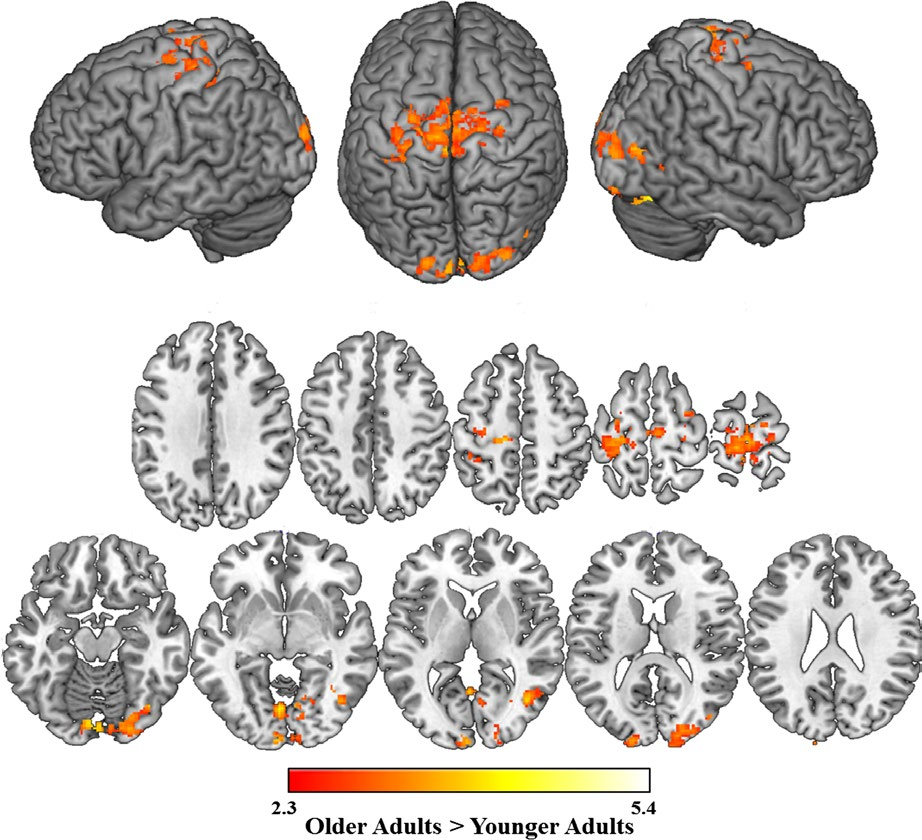
Age differences in the effect of target frequency. Results of a parametric analysis in which increases in activation were associated with decreases in target frequency. Older adults displayed greater modulation of activation than younger adults during naming with phonological distractors compared to nonword distractors. Slices are depicted in increments of 10, starting at z = −15 and ending at z = 75

Parametric analyses examined brain activation that increased with decreasing target frequency (see Table 5). Older adults displayed increases in activation in the bilateral precentral gyrus, middle supplementary motor cortex, and right occipital cortex associated with decreasing target frequency when targets were presented with phonological distractors as compared to nonword distractors. This activation was significantly greater for older compared to younger adults (see Figure 6).

**Table 5:** Parametric modulation of brain activation with decreasing target frequency

	H	Coordinates			Voxels	z value
		x	y	z		
Phonological > Nonword						
Younger Adults						
No significant activation						
Older adults						
Precentral gyrus	Left	−10	−32	74	2035	3.64
Precentral gyrus	Right	18	−26	72		
Postcentral gyrus	Left	−36	−34	48		
Occipital pole	Right	8	−94	−12	1231	3.89
Lingual gyrus	Middle	−4	−74	−6		
Occipital fusiform gyrus	Right	26	−86	−18		
Occipital pole	Right	16	−94	10	886	3.84
Younger > Older						
No activation differences						
Older > Younger						
Precentral gyrus	Right	28	−6	64	24	3.01
Precentral gyrus	Right	30	−24	70	27	3.06
Postcentral gyrus	Middle	−6	−40	74	1272	3.61
Lateral occipital cortex	Right	44	−64	−12	364	3.97
Lateral occipital cortex	Right	20	−86	14	372	3.34
Lingual gyrus	Middle	−4	−74	−6	851	4.24
Occipital pole	Middle	−6	−98	−2	135	3.61
Occipital pole	Left	−16	−98	16	84	3.26
Unrelated > Nonword						
Younger Adults						
No significant activation						
Older Adults						
Frontal pole	Left	−28	42	−6	460	3.52
Occipital pole	Middle	6	−94	−10	4701	4.06
Lingual gyrus	Middle	−2	−74	−8		
Younger > Older						
No activation differences						
Older > Younger						
Frontal pole	Left	−34	52	−6	24	2.7
Frontal pole	Left	−42	46	0	80	2.88
Frontal pole/Frontal orbital cortex	Left	−30	40	−6	86	3.44
Lateral occipital cortex	Right	42	−66	−6	95	3.38
Lateral occipital cortex	Right	40	−72	14	86	3.02
Lateral occipital cortex	Right	44	−72	24	18	2.78
Lateral occipital cortex	Right	44	−80	30	10	2.99
Lateral occipital cortex	Left	−28	−90	−22	31	2.96
Lingual gyrus	Middle	−2	−82	−14	1246	3.6
Occipital pole	Right	18	−98	14	515	3.27
Nonword > Categorical						
Younger Adults						
Temporal pole	Right	28	10	−40	438	4.21
Inferior temporal gyrus	Right	46	2	−38		
Middle temporal gyrus	Right	46	−52	0	598	3.63
PHG/Hippocampus	Right	28	−40	0		
Older Adults						
PHG/Hippocampus	Right	36	−36	−6	26	3.16
Younger > Older						
Inferior temporal gyrus	Right	38	−54	−6	140	4.14
Nonword > Phonological						
Younger Adults						
Posterior cingulate	Middle	0	−40	2	677	3.94
Occipital fusiform gyrus	Right	44	−66	−20	862	3.97
Inferior temporal gyrus	Right	48	−56	−20		
Older Adults						
No significant activation						
Younger > Older						
Occipital fusiform gyrus	Right	42	−68	−18	487	4.23
Older > Younger						
No activation differences						

Older adults also exhibited increases in activation in the left frontal pole and bilateral occipital cortex that were associated with decreasing target frequency when targets were presented with unrelated distractors compared to nonword distractors. Age differences were observed, such that older adults showed greater modulation of activity in these regions compared to younger adults.

Younger adults exhibited increases in activation associated with decreasing target frequency in the right middle temporal gyrus, which extended into the hippocampus and parahippocampal gyrus, and the right temporal pole, which extended into the inferior temporal gyrus when distractors were nonwords compared to when they were categorically related to the target. Age differences revealed that decreasing target frequency modulated activation in younger adults to a greater extent than older adults in the right inferior temporal gyrus and parahippocampal gyrus, when the distractors were nonwords compared to categorically related to the target. Finally, younger adults also exhibited increased activation that was associated with decreasing target frequency in the middle posterior cingulate and right occipital fusiform gyrus when distractors were nonword compared to when they were phonologically related to the target. Decreasing target frequency modulated activation in the right occipital fusiform gyrus to a significantly greater extent for younger compared to older adults.

## 4 Discussion

The primary goal of this study was to better understand age‐related differences in language production during the presentation of distracting information. Such an investigation has the potential to lend insight into the roles of both transmission and inhibition deficits in language production. To this end, we hypothesized that transmission deficits would manifest as increases in activation in language processing regions during object naming in the presence of a categorical distractor for older adults compared to younger adults, while no age differences would be observed during object naming with phonological distractors. If language production is influenced by age‐related inhibition deficits, however, we expected age‐related reductions in inhibitory control regions, combined with neural differences in language control regions that reflect increased processing of both categorical and phonological distractors. The results of this study find partial support for the transmission deficit hypothesis and very little evidence for inhibition deficits. Each of the findings is discussed in detail below.

With respect to picture naming in the presence of categorical distractors compared to phonological distractors, older adults exhibited significantly more activation of the left middle frontal gyrus, bilateral precuneus, posterior cingulate, and left occipital cortex than younger adults. While these regions are not typically part of a core language network, they provide evidence that older adults processed the categorical distractors to a greater extent than younger adults. The left middle frontal gyrus has been associated with semantic control processing, displaying increased activation during semantic tasks relative to phonological tasks in both younger and older adults (Diaz, Johnson, et al., 2014) as well during both directed semantic organization (Savage et al., 2001) and after training on the use of such semantic strategies (Miotto et al., 2006). The current study extends these findings, demonstrating that older adults recruit this region to a greater extent than younger adults when presented with distracting information that is categorically related to the target. Relatedly, the precuneus and posterior cingulate have been included in a neuroanatomical model of semantic processing, potentially aiding in the integration of semantic and episodic memory processing (Binder & Desai, 2011). Considering the role of these regions, in the current study the presentation of a categorical distractor resulted in greater semantic processing, in particular in semantic control regions, for older adults than younger adults. This is consistent with previous accounts of the age‐related trajectory of the semantic network, which has been hypothesized to increase with age, suggesting that older adults will engage in more semantic elaboration or require more semantic integration (Taylor & Burke, 2002). Thus, although we did not observe age‐related differences in activation of regions that have previously been reported to be involved in semantic interference in younger adults (e.g., middle temporal gyrus and superior frontal gyrus), our results nevertheless indicate that older adults process categorical distractors to a greater extent than younger adults, employing regions outside the traditional language network.

With respect to picture naming in the presence of phonological distractors, older adults exhibited decreased activation relative to younger adults in the bilateral middle temporal gyrus, bilateral pre and postcentral gyri, right supramarginal gyrus, and bilateral precuneus. Neither younger nor older adults exhibited significant phonological facilitation at the behavioral level, which has been reported consistently in behavioral literature, but not as consistently in MRI studies of picture‐word interference (e.g., Abel et al., 2009; Diaz, Hogstrom, et al., 2014). Moreover, neither age group demonstrated priming‐related reductions in activation, which others have reported as decreases in activation in the superior temporal gyrus during the processing of phonological distractors relative to unrelated distractors (de Zubicaray & McMahon, 2009; de Zubicaray et al., 2002). Our results are more consistent with other recent studies (e.g., Abel et al., 2009, 2012; Diaz, Hogstrom, et al., 2014), as younger adults showed increased activation in regions including the middle temporal gyrus, superior temporal gyrus, and supramarginal gyrus, many of these regions have been previously associated with phonological processing (e.g., Vigneau et al., 2006). Additionally, portions of these activations extend to the ventral portion of the precentral gyrus, which has previously been associated with articulatory processes (Brown, Ngan, & Liotti, 2008; Brown et al., 2009; Takai, Brown, & Liotti, 2010). Our pattern of younger adult data support the emerging pattern that suggests that phonological processing associated with increases in activation are observed when target pictures are not repeated across the course of the experiment, while decreases in activation are more strongly linked to priming that may occur when target pictures are repeated. In this way, it will be important to continue exploring the independent contributions of phonological processing and priming. Older adults, however, did not show a similar pattern of activation. Rather, even though the phonological distractor was intended to aid in the retrieval of the target, older adults showed age‐related deficits in activation of regions including the middle temporal gyrus and supramarginal gyrus. The left middle temporal gyrus has been shown to support multiple aspects of language, including both phonological and semantic processing (Gernsbacher & Kaschak, 2003), while both the left (Church, Balota, Petersen, & Schlaggar, 2011) and right supramarginal gyrus (Diaz, Hogstrom, et al., 2014) has been demonstrated to be particularly sensitive to phonological processing. The results of the current study, which demonstrates that older adults recruit significantly less activation in regions that support phonological processing, are thus consistent with general declines in language production abilities that have been documented across a variety of different tasks (for review see: Diaz, Rizio, & Zhuang, 2016).

Our final set of analyses explored the effects of target frequency on age‐related differences in language production. Our results indicate that while both younger and older adults show the same pattern of negative correlation between target frequency and response latency, significant age differences were found when examining the effect of target frequency on brain activation. As target frequency decreased, older adults activated regions of the bilateral pre and postcentral gyrus to a greater extent than younger adults during phonological, as compared to nonword trials. These results indicate that providing access to the phonology of the targets disproportionally facilitated production of low‐frequency words. The precentral gyrus is part of the auditory‐motor speech coordination network, which supports phonological processing and speech production (Vigneau et al., 2006). However, these foci were more dorsal than the traditional articulatory homunculus (e.g., Brown et al., 2009), and more likely reflect multisensory integration (e.g., Butler & James, 2013; Chen, Zatorre, & Penhune, 2006) or perhaps an aspect of retrieval difficulty (e.g., Tan et al., 2008). Interestingly, others have found activation in this dorsal premotor region when young adults were asked to name difficult‐to‐name colors (e.g., nonprimary blues, shades of brown) compared to primary colors (Tan et al., 2008). As such, it appears as though providing access to the phonology of low‐frequency targets facilitated production to a greater extent for older than younger adults. Because the current study did not show that older adults were slowed by low‐frequency items to a greater extent than younger adults, it is possible that recruiting additional regions helped alleviate the effects of transmission deficits that would typically be observed for these items. Additional future work will need to focus on exploring the role of similar activation patterns in older adults who are more sensitive to frequency effects.

Overall, our findings are partially consistent with the theory that transmission deficits contribute to phonological age‐related differences in language production. Older adults exhibited neural evidence of increased semantic elaboration during the presentation of a categorical distractor. According to the TDH, the organization of the semantic system (i.e., it is highly interconnected and redundant, as compared to the phonological system) makes it less likely to be negatively affected by transmission deficits (MacKay & Burke, 1990). Our data support this aspect of the theory, in that age‐related reductions in activation related to language production were not observed when the distractor promoted semantic elaboration. Interestingly, while our neuroimaging results showed increased activation related to semantic organization for older adults while processing the categorical distractor, it did not appear to significantly interfere with their ability to name the target, as no age‐related differences were observed in response latencies. With respect to age‐related differences in phonological processing, we find partial evidence for the transmission deficit hypothesis. The presence of phonological distractors did not produce facilitatory behavioral effects. Instead, we observed neural evidence that older adults experience declines specifically in phonological processing, even when provided lexical cues to the target. Interestingly, these declines in activation did not result in observable behavioral differences in response latency between younger and older adults. Parametric analyses also support the notion that older adults experience transmission deficits during language production tasks, as we provide neural evidence that production of low‐frequency targets was aided by the phonological distractor to a greater extent than higher frequency targets. Older adults’ increased activation of the precentral gyrus for low‐frequency targets paired with phonological distractors suggests that facilitation can aid not only in lexical selection (as suggested by Taylor & Burke, 2002), but also in a later stage of language production, namely, at the level of articulation. Moreover, additional analyses indicated that it is unlikely that these effects are being driven by the frequency of the distractor words (see supplemental materials for these analyses). Our results suggest that because older adults experience transmission deficits that differentially affect low‐frequency items, providing access to their phonology increases the articulatory representation to a greater extent than for higher frequency items, which are not as affected by transmission deficits.

The current data provide little evidence that inhibition deficits contribute to age‐related differences in language production in the context of a PWI paradigm. The observed age differences in neural activity associated with semantic interference are not consistent with inhibition deficits, as there was no evidence that the two age groups differentially activated regions associated with inhibitory control. Moreover, we did not find evidence for inhibitory deficits during phonological processing, as the patterns of neural activation did not indicate that older adults processed the phonological distractors to a greater extent than younger adults. Behavioral evidence for the inhibition deficit theory was also scarce, as older adults were not more likely to read the distractor than younger adults. Instead, older adults showed a tendency to be more likely than younger adults to not provide any response at all, reflecting of the type of retrieval failure indicative of transmission deficits. Taken together, we did not find neural or behavioral evidence that older adults processed all distractors to a greater extent compared to younger adults.

As this was the first neuroimaging study to employ the picture‐word interference paradigm in older adults, limitations must be acknowledged. The lack of age differences in response latency to the target pictures was somewhat surprising. Results of our follow‐up study indicated that the younger adults who performed the PWI task in the scanner were significantly slower at target naming than younger adults who completed the same task at a computer, thereby eliminating any potential age differences in response latency. We tentatively speculate that the younger adults, who were generally quite accurate, slowed down their naming in an effort to reduce head motion. While all participants were trained to speak without moving their heads, the younger adults exhibited significantly less movement in the scanner than older adults, potentially because they slowed their speech rate. Despite this limitation, general slowing of the younger adults appears to be distributed evenly across all distractor conditions, thus not affecting the main effect of distractor condition. It is alternatively possible that rather than younger adults sacrificing speed for either increased accuracy or reduced head motion, older adults sped up their naming, resulting in the age‐related reduction in naming accuracy. While we cannot definitively rule this out, this seems less likely. When comparing younger adult response times to previously published data that employed a PWI paradigm in the scanner, it is clear that the young participants’ response latencies from the current study are much slower in comparison (e.g., Diaz, Hogstrom, et al., 2014; reported an average response latency across conditions of 956.75 ms, while Abel et al., 2009 reported an average response latency of 852.5 ms). The only previous study that used the PWI paradigm with older adults reported response latencies that were faster than those reported here (Taylor & Burke, 2002). Another study that incorporated picture naming without distractors reported that older adults average latency was 1227 ms (Obler et al., 2010) which is comparable to the present study. These findings, combined with the fact that previous work (Abel et al., 2009), as well as the current study, have demonstrated that younger adults show numerically longer response latencies while naming in the scanner as compared to naming outside the scanner, make it appear unlikely that this sample of older adults developed a strategy that would allow them to name *faster* in the scanner.

In conclusion, this study provides novel information regarding the neural correlates of age‐related differences in language production. Specifically, transmission deficits, rather than inhibition deficits, appear to be the primary cause of the observed differences in picture naming when presented with distracting information.

## Conflict of Interest

None declared.

## Note

1 The average overall error rate of 6.35% for younger adults may at first glance appear incorrect, given the reported accuracy rate of 75%. The error rate for younger adults is in fact 25%. Further dividing these by type, 6.96% were no responses, 10.31% acceptable alternatives, 7.75% were incorrect, and 0.40% were named distractors. These rates sum to 25%, but the number derived from the main effect of age in the error type analysis (6.35%) is an *average* of these rates. Thus, while the average percentage when collapsing across error types for younger adults is 6.5%, the percentage of total trials that were inaccurate is 25%.
