# Supplementary material for: Neural evidence for phonologically based language production deficits in older adults: An fMRI investigation of age‐related differences in picture‐word interference
Source: Brain Behav. 2017 Mar 15;7(4):e00660. doi: 10.1002/brb3.660 (PMC5390840; doi:10.1002/brb3.660)
Supplement: Supplementary file 1 [file BRB3-7-e00660-s001.docx]

**Supplemental Material**

*Effects of Target-Distractor Frequency Difference*

Although target and distractor word frequency were uncorrelated, it may be possible that distractor frequency, specifically the difference between the target picture frequency and distractor word frequency, influenced the patterns of brain activation. Additional parametric analyses examined brain activation that increased as a function of the difference between target and distractor frequency. No regions of activation were associated with increasing positive difference in frequency (i.e., target frequency greater than distractor frequency) in any contrast between distractor conditions.

When examining the negative difference between target and distractor frequency (i.e., distractor frequency greater than target frequency), younger adults showed modulation of brain activation in left hippocampus to a greater extent for unrelated distractors than for phonological distractors. No age differences were observed. Older adults showed modulation of brain activation as a function of the negative difference between target and distractor frequency in bilateral occipital cortex to a greater extent for unrelated distractors than nonword distractors. No age differences were observed.

*Brain-Behavior Correlations:*

A series of analyses explored the effect of behavioral covariates on differences in activation related to distractor conditions. Covariates included reaction time, no response rate, and scores on the Author Recognition test. None of these behavioral factors were shown to be significantly correlated with differences in activation between distractor conditions.

| **Supplemental Table 1: Response Latencies (ms)** | | | | |
| --- | --- | --- | --- | --- |
|  | **Categorical** | **Phonological** | **Unrelated** | **Nonword** |
|  | **Mean (SE)** | **Mean (SE)** | **Mean (SE)** | **Mean (SE)** |
| **Younger Adults** | 1417 (0.66) | 1301.5 (52.78) | 1328.5 (57.05) | 1310.5 (55.27) |
| **Older Adults** | 1437 (54.94) | 1345.5 (55.55) | 1361.5 (53.55) | 1381 (45.46) |

| **Supplemental Table 2: Regions of Activation in older and younger adults** | | | | | | | | | | | | |  |
| --- | --- | --- | --- | --- | --- | --- | --- | --- | --- | --- | --- | --- | --- |
|  | **H** | | **Coordinates** | | | | | | **voxels** | | **z value** | |  |
|  |  | | **x** | | **y** | | **z** | |  | |  | |  |
| **Categorical > Unrelated** |  | |  | |  | |  | |  | |  | |  |
| **Younger Adults** |  | |  | |  | |  | |  | |  | |  |
| Superior frontal gyrus | Right | | 26 | | 12 | | 58 | | 1031 | | 3.58 | |  |
| Middle frontal gyrus | Right | | 36 | | 8 | | 50 | |  | |  | |  |
| Posterior cingulate gyrus | Middle | | 2 | | -32 | | 46 | | 468 | | 3.45 | |  |
| Angular gyrus | Right | | 50 | | -46 | | 42 | | 2412 | | 3.52 | |  |
| Angular gyrus | Right | | 58 | | -54 | | 24 | |  | |  | |  |
| Precuneus | Middle | | -6 | | -68 | | 48 | |  | |  | |  |
| Supramarginal gyrus | Right | | 66 | | -42 | | 20 | |  | |  | |  |
| Lateral occipital cortex | Left | | -56 | | -62 | | 34 | | 785 | | 3.39 | |  |
| Supramarginal gyrus | Left | | -44 | | -46 | | 54 | |  | |  | |  |
| Angular gyrus | Left | | -54 | | -56 | | 44 | |  | |  | |  |
| **Older Adults** |  | |  | |  | |  | |  | |  | |  |
| Superior parietal lobe | Left | | -30 | | -52 | | 44 | | 436 | | 3.36 | |  |
| Precuneus | Right | | 10 | | -60 | | 42 | | 684 | | 3.29 | |  |
| Occipital fusiform gyrus, Cerebellum | Left | | -48 | | -72 | | -26 | | 518 | | 3.32 | |  |
|  |  | |  | |  | |  | |  | |  | |  |
| **Categorical > Phonological** |  | |  | |  | |  | |  | |  | |  |
| **Younger Adults** |  | |  | |  | |  | |  | |  | |  |
| *no activation* |  | |  | |  | |  | |  | |  | |  |
| **Older Adults** |  | |  | |  | |  | |  | |  | |  |
| Frontal pole | Left | | -22 | | 40 | | 46 | | 1313 | | 4.01 | |  |
| Middle frontal gyrus | Left | | -44 | | 20 | | 46 | |  | |  | |  |
| Superior frontal gyrus | Left | | -22 | | 34 | | 40 | |  | |  | |  |
| Precuneus | Right | | 10 | | -50 | | 60 | | 664 | | 3.22 | |  |
| Posterior cingulate gyrus | Middle | | -4 | | -36 | | 28 | |  | |  | |  |
| Cuneus | Right | | 8 | | -78 | | 40 | |  | |  | |  |
| Precuneus | Left | | -12 | | -54 | | 52 | | 638 | | 3.4 | |  |
| Postcentral gyrus | Left | | -32 | | -36 | | 70 | |  | |  | |  |
| Superior parietal lobe | Left | | -16 | | -52 | | 60 | |  | |  | |  |
| Lateral occipital cortex | Left | | -32 | | -66 | | 6 | | 582 | | 3.54 | |  |
| Temporal occipital fusiform cortex | Left | | -38 | | -48 | | -4 | |  | |  | |  |
| Occipital fusiform gyrus | Left | | -48 | | -70 | | -24 | | 3045 | | 3.74 | |  |
| Cerebellum | Left | | -50 | | -54 | | -32 | |  | |  | |  |
| Lingual gyrus | Right | | 8 | | -68 | | -10 | |  | |  | |  |
| Lingual gyrus | Middle | | -4 | | -70 | | -4 | |  | |  | |  |
| **Phonological > Unrelated** | |  | |  | |  | |  | |  | |  | |
| **Younger Adults** | |  | |  | |  | |  | |  | |  | |
| Angular gyrus | | Left | | -54 | | -54 | | 42 | | 21737 | | 4.39 | |
| Angular gyrus | | Right | | 52 | | -56 | | 52 | |  | |  | |
| IFG, pars triangularis | | Left | | -50 | | 36 | | 0 | |  | |  | |
| Middle frontal gyrus | | Left | | -34 | | 16 | | 56 | |  | |  | |
| Middle frontal gyrus | | Right | | 38 | | 8 | | 50 | |  | |  | |
| Superior frontal gyrus | | Left | | -26 | | -2 | | 64 | |  | |  | |
| Supplementary motor cortex | | Middle | | 2 | | 0 | | 52 | |  | |  | |
| Putamen | | Left | | -28 | | -12 | | 4 | |  | |  | |
| Insula | | Left | | -36 | | -12 | | 14 | |  | |  | |
| Central opercular cortex | | Right | | 54 | | -18 | | 16 | |  | |  | |
| Precentral gyrus | | Right | | 44 | | -14 | | 58 | |  | |  | |
| Precentral gyrus | | Left | | -14 | | -26 | | 64 | |  | |  | |
| Postcentral gyrus | | Left | | -62 | | -22 | | 26 | |  | |  | |
| Postcentral gyrus | | Right | | 48 | | -30 | | 50 | |  | |  | |
| Middle temporal gyrus | | Left | | -54 | | -30 | | -8 | |  | |  | |
| Middle temporal gyrus | | Right | | 66 | | -48 | | 4 | |  | |  | |
| Inferior temporal gyrus | | Left | | -60 | | -34 | | -24 | |  | |  | |
| Supramarginal gyrus | | Left | | -52 | | -32 | | 48 | |  | |  | |
| Supramarginal gyrus | | Right | | 50 | | -44 | | 52 | |  | |  | |
| Superior parietal lobe | | Right | | 26 | | -46 | | 66 | |  | |  | |
| Superior parietal lobe | | Left | | -30 | | -56 | | 70 | |  | |  | |
| Precuneus | | Right | | 16 | | -64 | | 26 | |  | |  | |
| Lateral occipital cortex | | Right | | 24 | | -58 | | 70 | |  | |  | |
| Lateral occipital cortex | | Left | | -44 | | -84 | | 26 | |  | |  | |
| Occipital pole | | Right | | 32 | | -90 | | 28 | |  | |  | |
| **Older Adults** | |  | |  | |  | |  | |  | |  | |
| *no activation* | |  | |  | |  | |  | |  | |  | |
|  | |  | |  | |  | |  | |  | |  | |
| **Phonological > Categorical** | |  | |  | |  | |  | |  | |  | |
| **Younger Adults** | |  | |  | |  | |  | |  | |  | |
| Supamarginal gyrus | | Right | | 50 | | -28 | | 44 | | 3202 | | 3.82 | |
| Precentral gyrus | | Right | | 54 | | 2 | | 22 | |  | |  | |
| Postcentral gyrus | | Right | | 62 | | -12 | | 22 | |  | |  | |
| Insula | | Right | | 36 | | -8 | | 12 | |  | |  | |
| Central opercular cortex | | Right | | 38 | | -10 | | 22 | |  | |  | |
| Postcentral gyrus | | Left | | -54 | | -18 | | 30 | | 3060 | | 3.79 | |
| Precentral gyrus | | Left | | -54 | | 8 | | 28 | |  | |  | |
| Precentral gyrus/IFG, pars opercularis | | Left | | -48 | | 4 | | 26 | |  | |  | |
| Putamen | | Left | | -30 | | -6 | | -2 | |  | |  | |
| Central opercular cortex | | Left | | -56 | | -16 | | 20 | |  | |  | |
| Lingual gyrus | | Right | | 14 | | -62 | | -2 | | 942 | | 3.41 | |
| Intracalcarine cortex | | Right | | 16 | | -62 | | 8 | |  | |  | |
| Precuneus | | Right | | 10 | | -62 | | 16 | |  | |  | |
| Cuneus | | Right | | 16 | | -68 | | 20 | |  | |  | |
| Superior lateral occipital cortex | | Right | | 18 | | -76 | | 40 | |  | |  | |
| Cuneus | | Left | | -16 | | -78 | | 34 | | 503 | | 3.9 | |
| Precuneus | | Left | | -14 | | -70 | | 24 | |  | |  | |
| Superior lateral occipital cortex | | Left | | -12 | | -78 | | 44 | |  | |  | |
| **Older Adults** | |  | |  | |  | |  | |  | |  | |
| *no activation* | |  | |  | |  | |  | |  | |  | |
